# Supplementary material for: Evaluation of Electrochemical Stability of Sulfonated Anthraquinone-Based Acidic Electrolyte for Redox Flow Battery Application
Source: Molecules. 2021 Apr 24;26(9):2484. doi: 10.3390/molecules26092484 (PMC8123158; doi:10.3390/molecules26092484)
Supplement: Supplementary file 1 [file molecules-26-02484-s001.zip › molecules-1185146-supplementary.pdf]

Supplementary Materials to

Article

Evaluation of Electrochemical Stability of Sulfonated Anthraquinone-Based Acidic Electrolyte for Redox Flow Battery Application

Petr Mazúr [1,2,*], Jiří Charvát [1], Jindřich Mrlík [1], Jaromír Pocedič [2], Jiří Akrman [3], Lubomír Kubáč [3], Barbora Řeháková [4] and Juraj Kosek [1,2]

1 University of Chemistry and Technology, Prague, Technická 5, 166 28 Praha 6, Czech Republic; mazurp@vscht.cz (P.M.); charvatj@vscht.cz (J.C.); mrlikj@vscht.cz (J.M.); jkk@vscht.cz (J.K.)
2 New Technologies—Research Centre, University of West Bohemia, Univerzitní 8, 306 14 Plzeň, Czech Republic; pocedicj@ntc.zcu.cz (J.P.)
3 Centre for Organic Chemistry, Rybitvi 296, 533 54 Rybitvi, Czech Republic; jiri.akrman@cocltd.cz (J.A.); lubomir.kubac@cocltd.cz (L.K.)
4 Synthesia, a.s., Semtín 103, 530 02 Pardubice, Czech Republic; Barbora.Rehakova@synthesia.cz (B.Ř.)
* Correspondence: mazurp@vscht.cz

List of content

Figure S1: HPLC chromatogram of anthraquinone sulfonation mixture after synthesis.

Figure S2: UV-vis spectrum of initial electrolyte and electrolyte after electrochemical test, both samples diluted 50 000 times by demineralized water, measured in cuvette with the optical length of 1 cm.

Figure S3: Equivalent circuit used for evaluation of EIS spectra of RFB full-cell consisting of: R1 – ohmic resistance of the cell, R2 and R3 – charge transfer resistance of both electrodes, Q2 and Q3 – constant phase elements representing electrode-electrolyte interface on both interfaces.

Table S1: Composition of the AQDS negolyte before and after electrochemical test in the redox flow battery obtained by HPLC

Table S2: Composition of AQDS electrolyte after 5 days in different SOC and temperatures

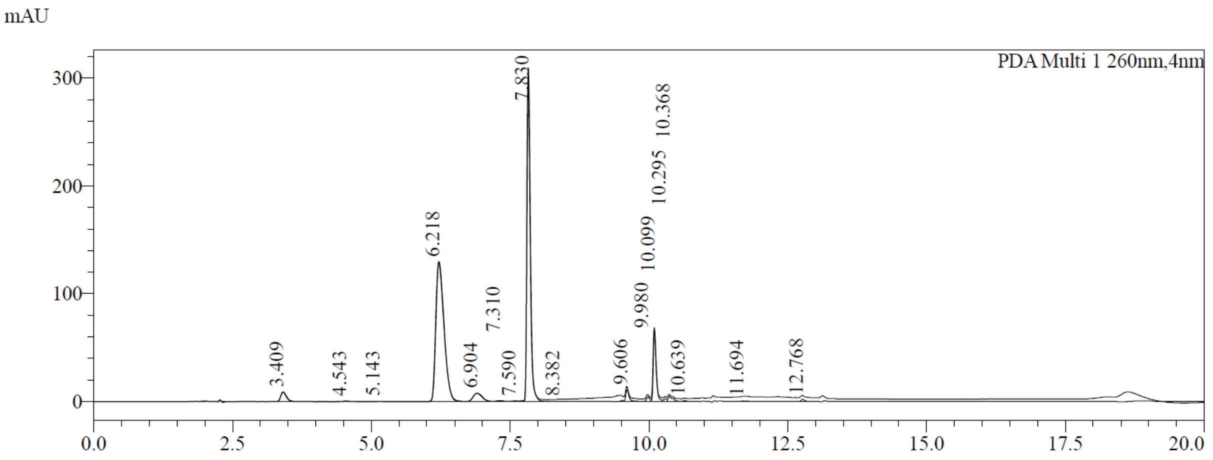

Figure S1: HPLC chromatogram of anthraquinone sulfonation mixture after synthesis.

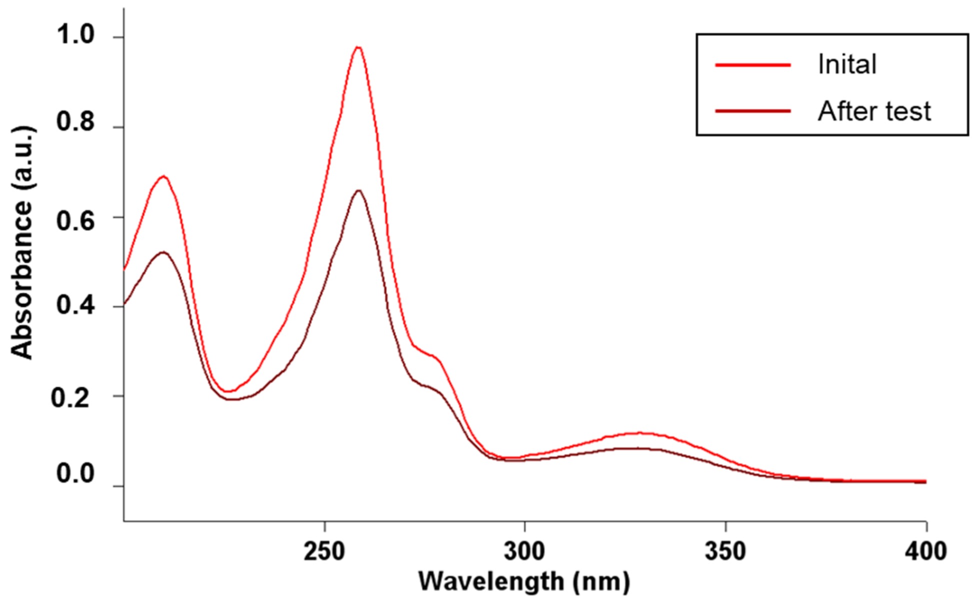

Figure S2: UV-vis spectrum of initial electrolyte and electrolyte after electrochemical test, both samples diluted 50 000 times by demineralized water, measured in cuvette with the optical length of 1 cm.

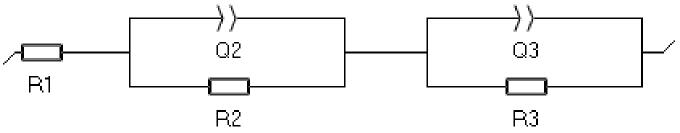

Figure S3: Equivalent circuit used for evaluation of EIS spectra of RFB full-cell consisting of: R1 – ohmic resistance of the cell, R2 and R3 – charge transfer resistance of both electrodes, Q2 and Q3 – constant phase elements representing electrode-electrolyte interface on both interfaces.

Table S1: Composition of the AQDS negolyte before and after electrochemical test in the redox flow battery obtained by HPLC

Compound	Retention time (min)	Initial electrolyte (area %)	Electrolyte after test (area %)
**anthraquinone-1,7-disulfonic acid**	3.4	2.1	2.0
**anthraquinone-2,6-disulfonic acid**	6.2	41.9	44.1
**anthraquinone-1,6-disulfonic acid**	6.9	2.8	3.0
**unknown compound - 1**	7.3	0.2	0.3
**anthraquinone-2,7-disulfonic acid**	7.8	41.0	43.0
**unknown compound - 2**	9	0.0	0.4
**unknown compound - 3**	9.4	0.0	0.3
**anthraquinone-1-sulfonic acid**	9.6	1.5	0.3
**2-hydroxyanthraquinone-1-sulfonic acid**	10.0	0.6	0.2
**anthraquinone-2-sulfonic acid**	10.1	7.7	3.2
**unknown compound - 4**	10.4	0.8	0.8

Table S2: Composition of AQDS electrolyte after 5 days in different SOC and temperatures

**SoC / %**	**0**	**0**	**25**	**50**	**50**	**75**	**75**	**85**	**95**	**100**	**100**
**Temperature / °C**	**20**	**60**	**60**	**20**	**60**	**20**	**60**	**60**	**60**	**20**	**60**
c AQDS (1)/ mol dm^{-3}	0.84	0.85	0.82	0.81	0.81	0.80	0.72	0.68	0.68	0.79	0.65
c H_2SO_4 (2)/ mol dm^{-3}	1.14	1.36	1.03	1.04	1.09	1.06	1.27	1.31	1.17	1.02	1.16
c total sulphate / mol dm^{-3}	2.82	3.06	2.67	2.66	2.71	2.66	2.71	2.67	2.53	2.60	2.46
	**Content (3) / Area %**										
anthraquinon-1,7-disulfonic acid	1.80	1.83	1.80	1.78	1.40	1.79	1.34	1.28	1.31	1.83	1.29
anthraquinon-2,6-disulfonic acid	39.15	39.30	41.28	39.20	40.11	39.54	41.70	42.14	43.95	41.49	43.96
anthraquinon-1,6-disulfonic acid	1.91	1.97	2.66	2.37	2.14	2.06	1.73	1.92	1.69	2.06	2.06
anthraquinon-2,7-disulfonic acid	43.37	43.22	42.49	42.85	43.39	43.00	44.10	44.73	45.66	44.67	45.85
anthraquinon-1-sulfonic acid	2.06	2.07	1.55	2.06	1.44	1.97	0.76	0.62	0.50	1.14	0.55
anthraquinon-2-sulfonic acid	11.72	11.61	10.22	11.74	11.53	11.65	10.38	9.31	6.90	8.82	6.30
2,6-AQDS / 2,7-AQDS / area % ratio	0.90	0.91	0.97	0.91	0.92	0.92	0.95	0.94	0.96	0.93	0.96
2,6- and 2,7-AQDS / area % ratio	0.83	0.83	0.84	0.82	0.83	0.83	0.86	0.87	0.90	0.86	0.90

(1)UV-vis spectrophotometry, (2)acido-basic titration by NaOH, (3)HPLC
